# An enhanced adaptive elephant herding optimization based on hybrid cuckoo search algorithm and elite opposition-based learning

**DOI:** 10.1038/s41598-026-48615-y

**Published:** 2026-05-16

**Authors:** Zahraa Elsayed Mohamed, Walid Dabour

**Affiliations:** 1 Department of Mathematics, Faculty of Science, Zagazig University, P.O. Box 44519, Zagazig, Egypt; 2 Department of Mathematics and Computer Science, Faculty of Science, Menoufia University, Shebin El Kom, Menofia 32511 Egypt; 3 Menoufia National University, MNU, Cairo - Alexandria Agricultural Road, 70 KM, Menoufia, Egypt

**Keywords:** Meta-heuristic algorithm, elephant herding optimizer, cuckoo search algorithm, elite opposition-based learning, adaptive approach, hybriding technique, Engineering, Mathematics and computing

## Abstract

This study proposes a hybrid variant of Elephant Herding Optimization that combines an adaptive clan-updating schedule, Lévy-flight exploration from Cuckoo Search, and elite opposition-based sampling. The design targets three known limitations—premature convergence, imbalance between exploration and exploitation, and slow late-stage progress—by (i) nonlinearly annealing clan influence across iterations, (ii) applying long-range moves exclusively to clan leaders, and (iii) injecting diversity around elite candidates. On ten standard benchmarks, the proposed method achieves faster convergence and lower error than EHO, PSO, SCA, and EHO-based hybrids.For example, it reaches the global optimum on F6 and attains a mean error on F1 that is 12.8× lower than that of PSO under identical evaluation budgets. A signal-processing case study (Wiener spline filter design) further demonstrates transferability to an industry-relevant task. We analyze parameter sensitivity, provide a time-complexity breakdown, and discuss limitations. The approach offers a systematic and generalizable hybrid framework that balances broad exploration early with precise exploitation late.

**Supplementary Information:**

The online version contains supplementary material available at 10.1038/s41598-026-48615-y.

## Introduction

Metaheuristic algorithms have emerged as powerful tools for solving complex optimization problems due to their ability to efficiently explore large search spaces^1^. These algorithms can be classified into four main categories: evolutionary-based, physics-based, swarm intelligence, and human-based methods. Evolutionary-based algorithms, inspired by Darwinian principles of natural selection, originated in the early 1970s and utilize genetic operators such as crossover, mutation, and selection to evolve solutions over generations^2^. Prominent examples include Genetic Algorithms (GA)^3^, Biogeography-Based Optimization (BBO)^4^, and Differential Evolution (DE)^5^. Physics-based algorithms, on the other hand, model optimization processes after physical phenomena, with notable implementations including the Equilibrium Optimizer (EO)^6^ and Gravitational Search Algorithm (GSA)^7^.

Swarm intelligence algorithms mimic the collective behavior observed in natural systems, with widely used techniques such as the Slime Mould Algorithm (SMA)^8^, Cuckoo Search (CS)^9^, Salp Swarm Algorithm (SSA)^10^, Particle Swarm Optimization (PSO)^11^, and Ant Colony Optimization (ACO)^12^. Human-based algorithms, though less common, simulate human decision-making processes, as exemplified by the Harmony Search (HS) algorithm^13^. Among these, the Elephant Herding Optimization (EHO) algorithm, introduced by Wang et al. in 2015^14^, stands out for its unique inspiration drawn from the social hierarchy of elephant herds. The algorithm operates through two primary mechanisms: clan updating, where individuals move toward the clan’s best member, and separation, which reinitializes poorly performing individuals to maintain population diversity.

Despite its innovative approach, EHO suffers from several limitations, including slow convergence, an imbalance between exploration and exploitation, and a tendency to become trapped in local optima. These drawbacks stem from the algorithm’s inherent stochastic nature and rigid parameter settings. To address these issues, researchers have proposed various enhancements. Muthusamy et al.^15^ hybridized EHO with the Sine Cosine Algorithm (SCA) to improve convergence rates, while Li and Wang^16^ incorporated dynamic topology and Biogeography-Based Optimization (BBO) techniques to refine the algorithm’s operators. Ismaeel et al.^17^ developed some unbiased algorithm variations to enhance EHO’s capacity for global optimization. Elhosseini et al.^18^ suggested some similar distinctions: cultural-based, alpha-tuning, and biased initialization EHO. Whereas, Balamurugan et al.^19^ introduced a technique to improve EHO’s performance, and Xu et al.^20^ integrated Lévy flight into EHO (LFEHO) to mitigate premature convergence. Additionally, Suganyadevi et al.^21^ combined chaotic maps with EHO and Convolutional Neural Networks (CNN) to improve detection accuracy in specific applications.

However, many of these modifications still struggle with maintaining population diversity and avoiding local optima. To overcome these persistent challenges, this study proposes AEHOCSEOBL, a novel hybrid algorithm that synergizes three key components: Adaptive EHO (AEHO), Cuckoo Search (CS), and Elite Opposition-Based Learning (EOBL). AEHO introduces dynamic parameter control to enhance flexibility and search efficiency, while CS strengthens global exploration capabilities. EOBL further improves performance by expanding the search space and preventing stagnation in local optima. Recent advancements in hybrid metaheuristics confirm that combining multiple complementary strategies can effectively overcome individual algorithm limitations. For example, Nayak et al. (2024) demonstrated that an elitist opposition-based artificial electric field algorithm significantly improves forecasting accuracy in financial time series by enhancing population diversity^22^. Similarly, Wang and Zhang (2025) showed that dynamic opposition learning integrated into a teaching–learning optimizer yields more reliable parameter extraction for photovoltaic models^23^. These studies underscore the value of opposition-based and adaptive mechanisms for balancing exploration and exploitation. Motivated by these findings, our work integrates adaptive clan updating, Cuckoo Search’s Lévy flights, and elite opposition-based learning within a unified EHO framework.

Recent studies further highlight the value of hybrid and opposition-enhanced metaheuristics for challenging engineering objectives. In signal processing, evolutionary design of Wiener spline nonlinear adaptive filters demonstrated the need for optimizers that balance global search with fine-grained local refinement^24^. Within the EHO family, gradient-based enhancements improved cluster analysis by stabilizing updates around informative directions, indicating that scheduled exploitation can elevate baseline EHO performance^25^. Opposition mechanisms, such as lens opposition in swarm hybrids, consistently boost diversity and help escape local minima^26^. Adaptive Cuckoo Search hybrids tailored for industrial engineering achieve stronger convergence without sacrificing robustness^27^, and artificial electric field schemes combined with Cuckoo Search and refraction learning report competitive results on constrained tasks^28^. Together, these trends motivate our tri-component design—adaptive EHO dynamics, Lévy-flight exploration, and elite opposition—as a principled strategy for achieving early broad coverage and late precise convergence.

The primary contributions of this work are:

1. A novel hybrid algorithm, AEHOCSEOBL, that synergistically combines adaptive EHO, Cuckoo Search, and elite opposition-based learning within a unified framework.
2. An adaptive clan updating mechanism with nonlinear dynamic parameter control to balance exploration and exploitation throughout the optimization process.
3. Integration of elite opposition-based learning to enhance population diversity and prevent stagnation in local optima.
4. Comprehensive validation of the proposed method on ten benchmark functions and two real-world engineering problems (Wiener spline filter design and welded beam design).

The primary contributions of this work are threefold. First, we present AEHOCSEOBL as an enhanced version of the original EHO algorithm. Second, we implement an adaptive clan updating mechanism to improve convergence and exploration through dynamic parameter adjustments. Third, we integrate CS and EOBL to bolster global search performance and escape local optima, respectively. The proposed algorithm is rigorously evaluated using ten benchmark functions and compared against established methods such as EHO, PSO, and CS. Experimental results demonstrate that AEHOCSEOBL outperforms these alternatives in terms of solution accuracy, convergence speed, and stability.Beyond benchmark optimization, the proposed algorithm is well-suited for real-world applications where balanced exploration–exploitation and robustness are critical, including renewable energy system design, neural network training, and supply chain optimization. The approach is particularly attractive for black-box, noisy, or non-convex objectives, such as: (i) engineering design with expensive simulations (e.g., aerodynamic or structural shape optimization), (ii) machine learning hyperparameter tuning in mixed discrete–continuous spaces, and (iii) photovoltaic parameter extraction, where local minima and measurement noise are common.

*Novelty* We introduce a unified update schedule that fundamentally differs from prior EHO hybrids: (i) it couples EHO’s clan dynamics with a nonlinear adaptive control parameter α that deterministically anneals the transition from exploration to exploitation; (ii) it injects Lévy-flight updates exclusively to matriarchs, enabling controlled long-range relocations of the search center without destabilizing followers; and (iii) it applies elite opposition-based learning around the current top-k solutions to deliberately expand—and later contract—the high-quality basin. This sequential, mechanism-level balance is absent in previous EHO hybrids, which typically add a single mechanism (e.g., OBL or SCA) uniformly to all agents.

In summary, the key technical contributions are:

1. a triple‑hybrid schedule (AEHO and matriarch‑only CS and elite‑window EOBL) with a deterministic adaptation law;
2. a matriarch‑centric Lévy step that repositions clan leaders without destabilizing followers;
3. an elite opposition layer that provably increases population variance near elites (Sect.  3.3) while preserving feasibility; and.
4. comprehensive evaluation on ten benchmarks plus two real‑world case studies, including ablation experiments isolating each mechanism.

The remainder of this paper is organized as follows. Section  2 provides a detailed review of the classical EHO and CS algorithms. Section  3 elaborates on the proposed methodology, including the adaptive mechanisms and hybridization strategies. Section  4 presents the experimental results and comparative analysis. Finally, Sect.  5 concludes the study and suggests directions for future research.

## Preliminary

### EHO

The Elephant Herding Optimization (EHO) algorithm, developed by Wang et al. in 2015^14^, draws inspiration from the social hierarchy and herding behavior observed in elephant populations. The algorithm begins by dividing the initial population into i distinct clans, each containing X individuals. Within each clan, the individual with the best fitness value is designated as the matriarch, serving as the leader and reference point for other clan members.

During the optimization process, each clan member updates its position to move closer to the matriarch’s location, effectively improving its standing within the clan hierarchy. This position update mechanism represents the algorithm’s exploitation phase, where solutions are refined based on local information. To maintain population diversity and prevent premature convergence, the algorithm implements a separation operator that replaces the worst-performing individuals in each clan with new randomly generated solutions at the end of every iteration.

The EHO algorithm operates through two fundamental mechanisms: the clan updating operator and the separation operator. The clan updating operator guides individuals toward their respective matriarchs, facilitating local refinement of solutions. Subsequently, the separation operator reintroduces diversity by eliminating and replacing the least fit individuals. This cyclical process of position updates followed by selective replacement continues iteratively until either the optimal solution is found or the maximum number of iterations is reached. The balanced application of these two operators enables EHO to maintain an effective equilibrium between exploitation of known good solutions and exploration of new regions in the search space.

#### Clan update operator

In the EHO algorithm, each clan operates under the leadership of its matriarch, with all clan members coexisting and evolving collectively. The matriarch’s position serves as a guiding influence for all elephants within its clan, directing their movement and position updates. The position update for elephant j in clan i can be mathematically expressed as follows:

1 $$\:{x}_{i,j}^{new}={x}_{i,j}+\:\alpha\:\:\times\:\left({x}_{best,j}-\:{x}_{i,j}\right)\times\:\:r$$

where $\:{x}_{i,j}^{new}$ and $\:{x}_{i,j}$ represent the newly updated and old locations for elephant j in clan i, respectively. Matriarch i’s influence on $\:{x}_{i,j}$ is determined by a scale factor called α is between 0 and 1, where $\:{x}_{best,j}$ stands for matriarch i, the best elephant individual in clan i was $\:{x}_{best,j}$ and r $\:\in\:$ [0, 1]. The following formula in Eq. (2) can be used to update which elephant in each clan i is the best:

2 $$\:{x}_{i,j}^{new}={\upbeta\:}\:\times\:\left({x}_{center,i}\right)$$

where β $\:\in\:$ [0, 1] is a component that establishes the impact of$\:{\:x}_{center,i}$ on $\:{x}_{i,j}^{new}$.

Furthermore, $\:{\:x}_{center,i}$is the center of clan i, and it can be computed as follows in Eq. (3):

3 $$\:{x}_{center,i}=\frac{1}{{n}_{i}}\times\:\:\sum\:_{j=1}^{{n}_{i}}{x}_{i,j}$$

The number of elephants in clan i is denoted by $\:{n}_{i}$, and the individual elephant’s is denoted by $\:{x}_{i,j}$.

#### Separation operator

The following equation Eq. (4) proposes a separation operator to update the worst person in each clan i:

4 $$\:{x}_{iworst,i}^{t+1}={x}_{min}+\:\left({{x}_{max}-x}_{min}+1\right)\times\:r$$

The Elephant Herding Optimization algorithm operates within defined solution space boundaries, where x_max_ and x_min_ represent the upper and lower limits for each individual’s position respectively. A randomization factor r, uniformly distributed between [0,1], introduces stochastic exploration throughout the optimization process. The complete procedure, formally presented in Algorithm 1, incorporates these boundary constraints to maintain solution feasibility while enabling controlled exploration. The implementation features population initialization, clan organization with matriarch assignment, and systematic position updates that adhere to the boundary constraints. This computational framework preserves the algorithm’s biological inspiration while ensuring solution quality through its hierarchical update mechanisms and carefully balanced randomization. The pseudo-code explicitly outlines the processes of clan formation, position updates with boundary checking, and diversity maintenance, creating a robust optimization approach that prevents solution divergence while exploring the search space effectively.

**Algorithm 1:**
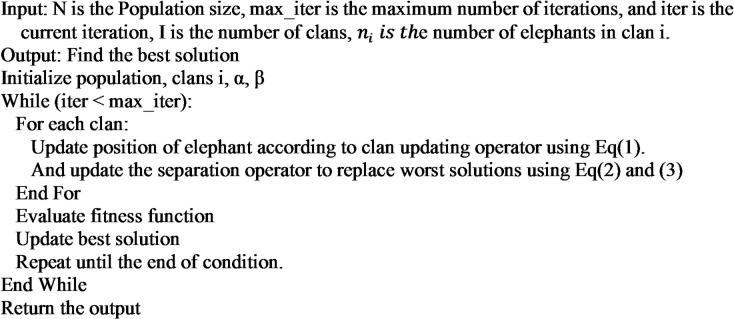
The pseudo-code for the main EHO.

### CSA

The Cuckoo Search Algorithm (CSA) is a meta-heuristic algorithm proposed by Yang and Deb in 2009 as a population-based stochastic optimization approach^29^. CSA employs Lévy flights for global search, with its random walks controlled by Lévy distributions. These distributions can occasionally generate large steps, enabling exploration of new regions in the search space and demonstrating powerful global search capabilities. As a result, CSA can search the designated space for optimal values more effectively than many other methods.

The algorithm is inspired by cuckoo birds, specifically their unique reproductive behavior and brood parasitism. In CSA, each cuckoo consistently lays one egg, representing a fundamental stage of the algorithm. Only the best nests containing superior eggs are carried forward to the next generation. The system operates with a fixed number of available host nests, where host birds have a probability Pa ∈ [0,1] of discovering the cuckoo’s egg. When this occurs, the host bird has two options: either discard the egg or abandon the nest to build a new one. Additionally, new nests can replace existing host nests with probability Pa. In CSA, Lévy flights are defined as shown in Eq. (5):

5 $$\:{x}_{i}^{t+1}={x}_{i}^{t}+\:\alpha\:\:{L}\acute{e}{vy}\left(\right)$$

where *α* is the step size and ⊕ denotes entry-wise multiplication. That, the Levy flight random path is more effective in searching the search space because the step of length is significantly greater over time. The following is an expression for a Levy flight based global exploratory random walk:

6 $${L}\acute{e}{vy} \:\sim\:\mathrm{u}={\mathrm{t}}^{-}\:,\:\:1<\le\:3$$

where λ denotes the mean of the event’s occurrence over a unit interval. A random path apply with a power law step-length distribution and a hefty tail is effectively formed by the steps in this case.

The typical CSA’s pseudo-code in Algorithm 2 is shown below:

**Algorithm 2:**
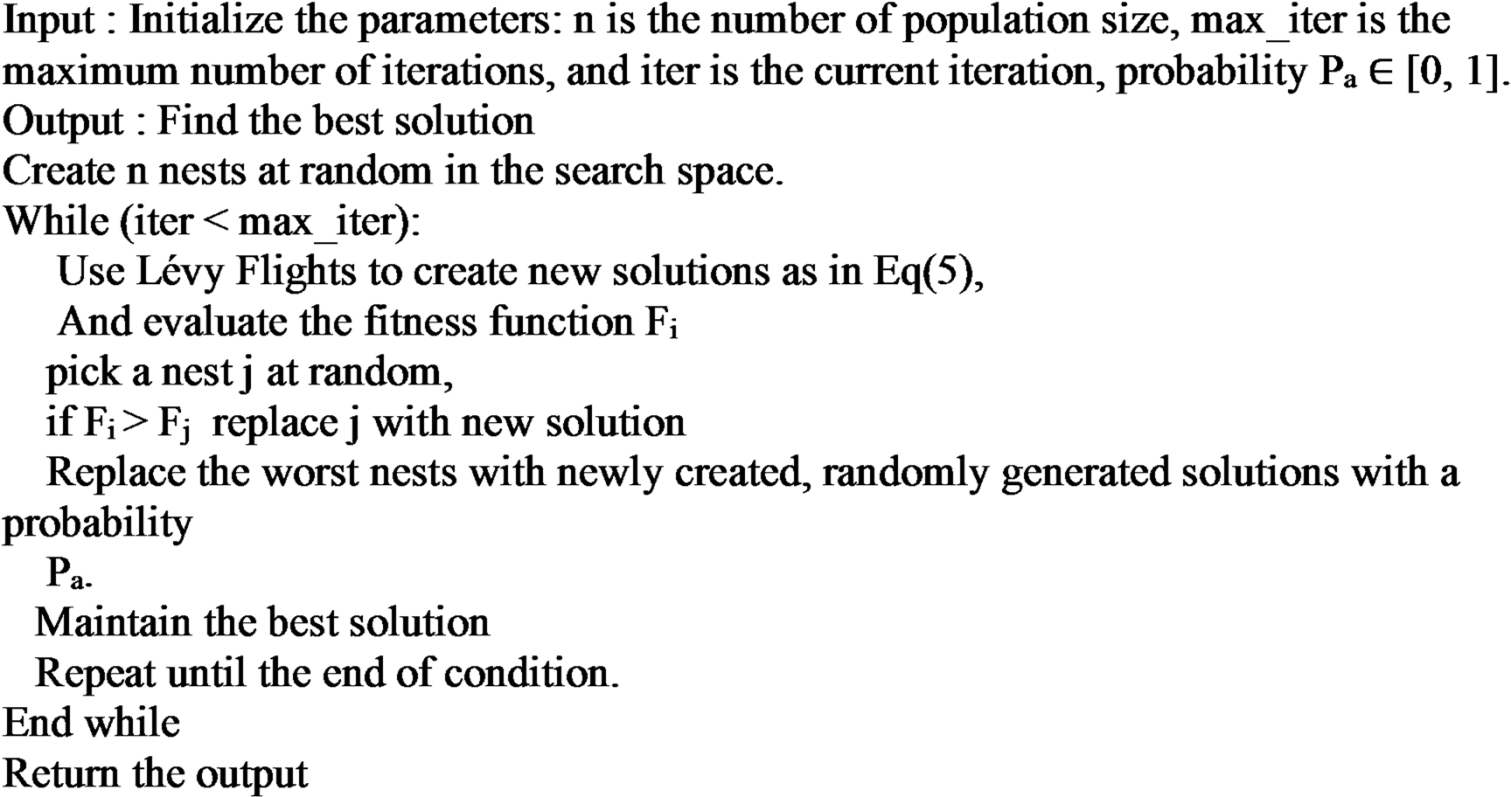
The pseudo-code for the main CSA.

## The proposed algorithm

The position update mechanism in EHO differs between clan leaders (matriarchs) and ordinary members. Each clan’s matriarch position significantly influences the optimization process, with its calculation varying based on specific algorithm parameters. However, this approach presents a critical limitation: without proper guidance toward globally optimal solutions, the algorithm may converge prematurely to local optima, compromising both exploration capability and convergence efficiency.

To address these limitations, this paper introduces three key enhancements to the basic EHO algorithm. First, we develop an Adaptive EHO (AEHO) variant that incorporates dynamic control parameters. These adaptive mechanisms automatically adjust during iterations to optimize position updates for all clan members, effectively balancing exploration and exploitation.

Second, we integrate the Cuckoo Search (CS) strategy through hybridization, leveraging its Lévy flight mechanism to enhance the original EHO’s search capabilities. This integration significantly improves the algorithm’s global optimization performance.

Third, we implement an Elite Opposition-Based Learning (EOBL) strategy during the exploitation phase. This addition substantially strengthens the algorithm’s ability to escape local optima traps while maintaining solution quality. Together, these innovations create a more robust and efficient optimization framework that overcomes the fundamental limitations of traditional EHO.

The combination of AEHO, CS, and EOBL is theoretically motivated by their complementary strengths: AEHO provides structured local exploitation through clan behavior but may converge prematurely; CS enhances global exploration via Lévy flights, yet its stochasticity can delay convergence; EOBL explicitly increases population diversity and helps escape local optima by generating elite-guided opposite solutions. Together, this triad addresses the limitations of each individual component—AEHO focuses exploitation, CS broadens exploration, and EOBL maintains diversity—resulting in a balanced and robust search strategy.

### The adaptive method

The adaptive control factor α plays a crucial role in maintaining equilibrium between global exploration and local exploitation throughout the optimization process. This dynamic parameter automatically adjusts its value during iterations, enabling the algorithm to progressively transition from broad exploration to focused exploitation. The nonlinear adaptation of α follows a carefully designed formulation Eq. (7) that ensures smooth and efficient search behavior evolution.

7 $$\alpha = 1 - 2/\left( {\exp ({\mathrm{iter}}/{\mathrm{Max}}\_{\mathrm{iter}})} \right)$$

As demonstrated in the results figures, this enables the algorithm to carry out extensive searches in the early iterations and adjust solutions as it converges.

### Hybrid adaptive method with CS

Recent advances in optimization have demonstrated the effectiveness of hybrid algorithms in solving complex real-world problems. By strategically combining complementary optimization techniques, these hybrid approaches achieve superior performance compared to standalone methods. However, the Elephant Herding Optimization (EHO) algorithm faces limitations when dealing with high-fitness individuals—its weak global search capability often leads to premature convergence in local optima.

To address this issue, we integrate the Cuckoo Search Algorithm (CSA) into EHO’s exploitation phase. Unlike EHO, CSA employs Lévy flight-based random walks, enabling more effective exploration of the search space. The Lévy distribution allows for occasional long jumps, facilitating transitions between distant regions and enhancing global search efficiency. This mechanism helps CSA identify optimal solutions more effectively than traditional methods.

In our hybrid approach, Lévy flights are utilized to update the positions of the best elephants (matriarchs) within each clan. This modification ensures that the matriarch’s position stabilizes with reduced fluctuations while progressively narrowing the distance between candidate solutions and the global optimum. By incorporating CSA, we introduce an additional set of search agents and dynamically refine existing positions, significantly improving EHO’s exploration-exploitation balance.

The accompanying flowchart illustrates how CSA-driven position updates enhance EHO’s core parameters. This hybridization not only strengthens control over the optimization process but also boosts the algorithm’s ability to solve challenging problems efficiently. The subsequent section details the proposed method’s workflow, supported by visual representation in the flowchart.

### EOBL

Opposition-based learning (OBL), first introduced by Tizhoosh^30^, has emerged as an effective optimization technique that accelerates convergence in metaheuristic algorithms^31–35^. The core principle of OBL involves evaluating both current solutions and their strategically generated opposites, then retaining the superior candidates. This approach has been successfully integrated into various optimization frameworks to improve solution quality and search efficiency.

In this work, we employ Elite Opposition-Based Learning (EOBL), an advanced variant that leverages elite population members to guide the search process. The rationale stems from the observation that elite individuals inherently possess more valuable information about promising search regions. EOBL operates by generating complementary opposite solutions relative to elite members within the defined search boundaries, as formalized in Eq. (8).

For a given individual X*X*, its elite opposite solution X′*X*′ is computed through a transformation that considers both the current elite population and the search space constraints. By simultaneously evaluating X*X* and X′*X*′, the algorithm retains the fitter candidate, ensuring progressive refinement toward optimal solutions. This mechanism not only enhances exploration but also prevents stagnation in local optima.

The mathematical formulation in Eq. (8) captures this opposition-based transformation, where the elite-guided approach provides a more informed search direction compared to standard OBL. Our integration of EOBL specifically targets the exploitation phase, where elite knowledge is most impactful for convergence.

8 $$\begin{aligned} {x_i}= & \left( {{x_{i,1}},{\text{ }}{x_{i,2}}, \ldots ,{\text{ }}{x_{i,d}}} \right),~\;x_{i}^{e}=\left( {x_{{i,1}}^{e},~x_{{i,2}}^{e}, \ldots ,x_{{i,d}}^{e}} \right),\;~x_{i}^{{e\_op}}=\left( {x_{{i,1}}^{{e\_op}},x_{{i,2}}^{{e\_op}}, \ldots ,x_{{i,d}}^{{e\_op}}} \right) \\ x_{{i,j}}^{{e\_op}}= & r \times \left( {lbj{\text{ }}+{\text{ }}ubj{\text{ }}} \right) - x_{{i,j}}^{e},\;\;\;i{\text{ }}=1,2, \ldots ,n{\text{ }},\;\;\;{\text{ }}j=1,2, \ldots ,d \\ \end{aligned}$$

Where $\:{\mathrm{x}}_{\mathrm{i},\mathrm{j}}^{\mathrm{e}\_\mathrm{o}\mathrm{p}}$ represents the elite opposite solution, lb_j_ and ubj are the lower and upper bound of the problem in j^th^ dimension, and r is a random number between 0 and 1, $\:{\mathrm{x}}_{\mathrm{i},\mathrm{j}}^{\mathrm{e}}$ represents the elite solution. n represents the total number of solutions; d represents the optimization problem’s dimension extend. The elite individual then directs the population to eventually arrive at the possible area where the global finest might be found. Thus, using the EOBL technique will improve the optimization algorithm’s global search and increase population diversity.

**Fig. 1:**
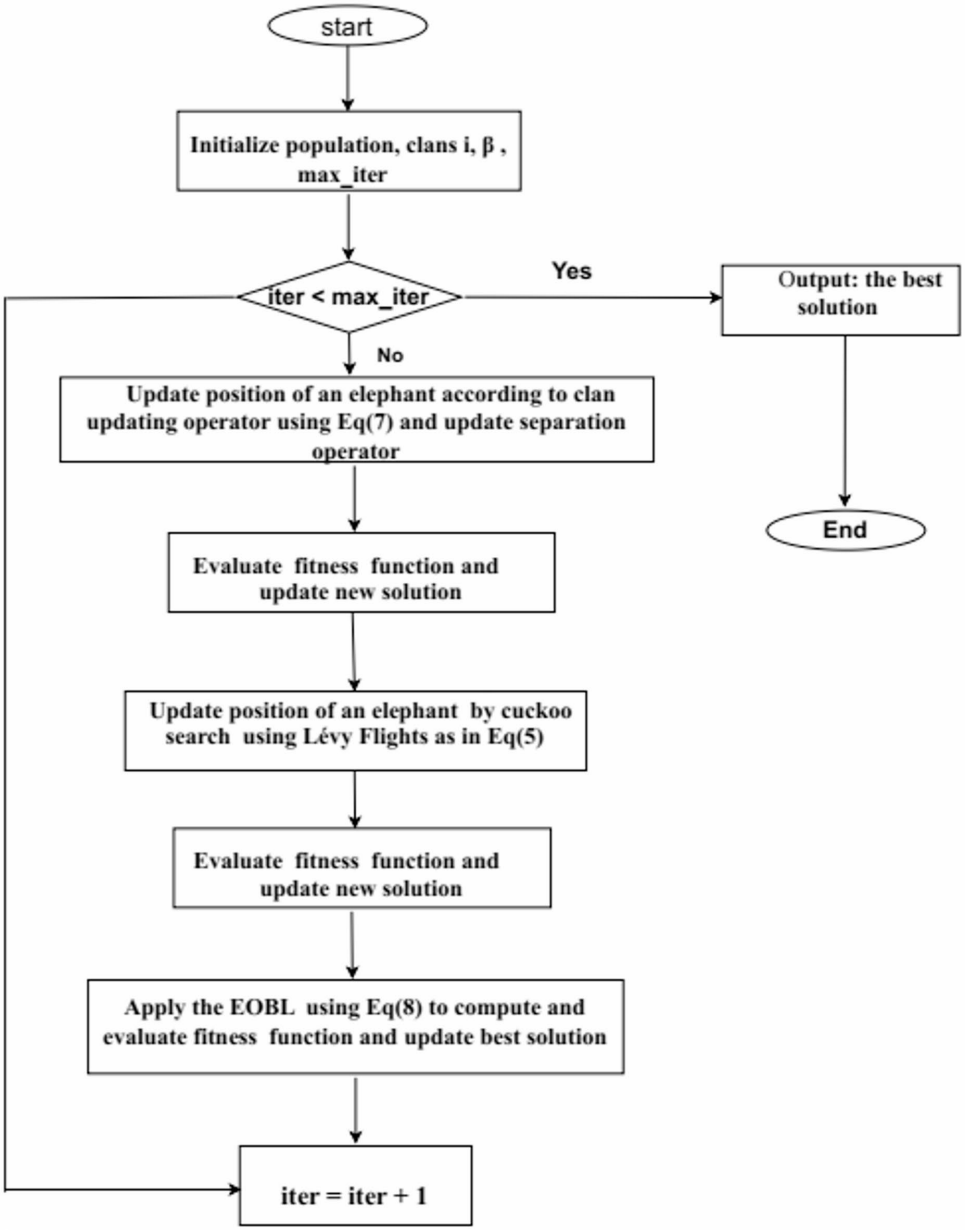
The flowchart of AEHOCSEOBL method.

The algorithm steps of proposed method as following in Algorithm 3 and the flowchart of improved method as in Fig. 1:

**Algorithm 3:**
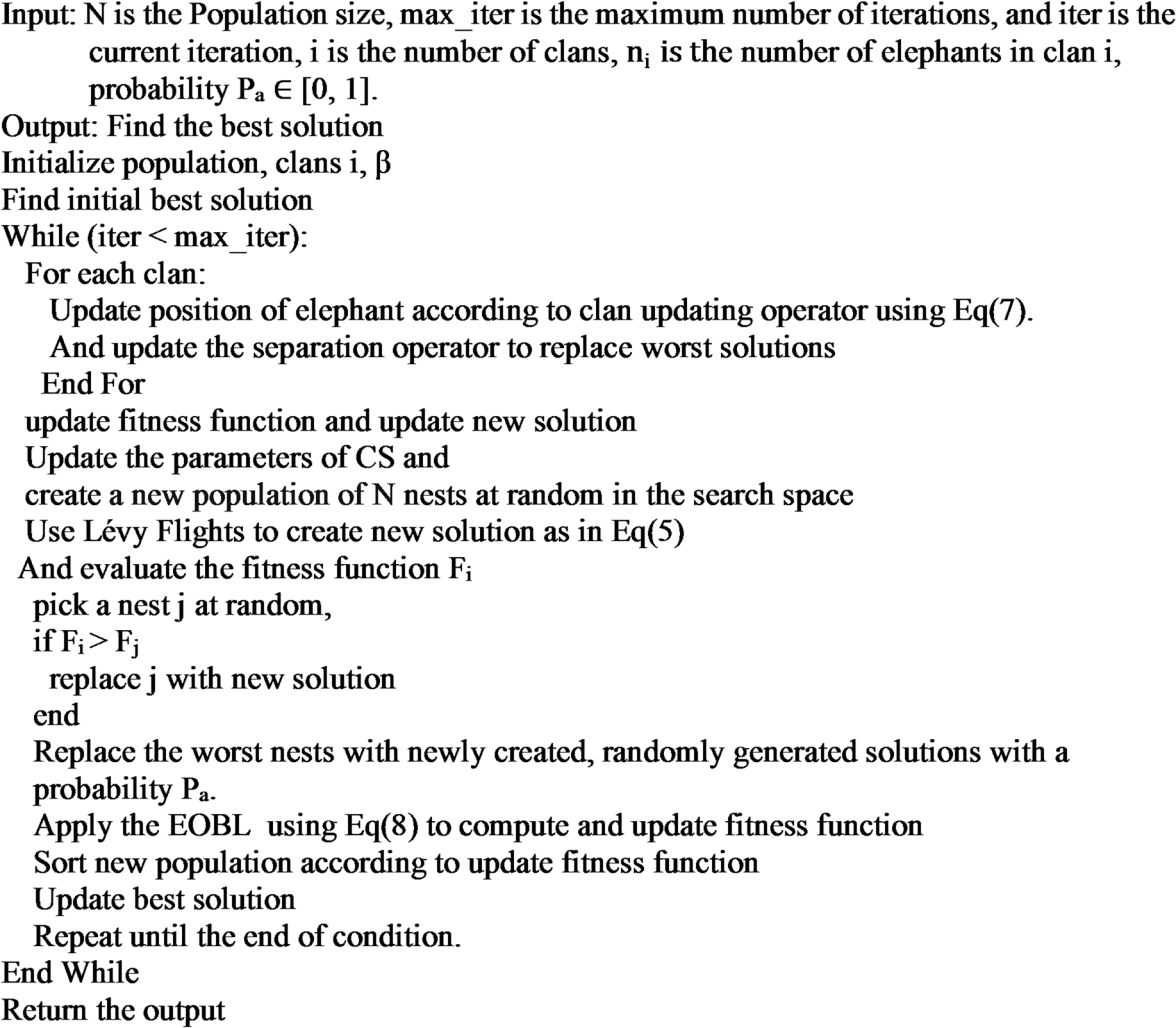
The pseudo-code for AEHOCSEOBL method.

Mechanistic rationale. Consider the population variance $\:{V}_{t}$ and the probability $\:{p}_{t}$ of sampling outside the current attraction basin. Lévy steps with exponent $\:\lambda\:\in\:\left(\mathrm{1,2}\right)$ produce heavy-tailed displacements whose tail probability decays as $\:O\left({\Delta\:}{x}^{-\lambda\:}\right)$, which increases $\:{p}_{t}$ for escaping deep basins compared to Gaussian perturbations. Applying Lévy flights only to matriarchs shifts basin centers without destabilizing followers, thereby preserving local exploitation. EOBL around elite individuals reflects candidates across elite-anchored centroids, provably increasing $\:{V}_{t}$ in the high-fitness region by a factor proportional to the elite spread; this counteracts the variance collapse typical in swarm updates. The adaptive parameter $\:\alpha\:\left(t\right)$ then deterministically contracts $\:{V}_{t}$ as iterations increase, providing a built-in annealing schedule. This “expand-then-contract” strategy yields a higher probability of hitting global basins early and faster local refinement later, compared to single-mechanism hybrids.

### Parameter selection and sensitivity

**Adaptive α schedule.** The nonlinear schedule.

The adaptive parameter $\:\alpha\:$ controls the influence of the clan’s best member during position updates. We propose the following nonlinear schedule:

$$\:\alpha\:\left(t\right)=1-\frac{2}{\mathrm{e}\mathrm{x}\mathrm{p}(t/{T}_{\mathrm{m}\mathrm{a}\mathrm{x}})}$$

where $\:t$ is the current iteration and $\:{T}_{\mathrm{m}\mathrm{a}\mathrm{x}}$ is the maximum number of iterations. This formulation yields $\:\alpha\:\approx\:0$ at $\:t=0$ (emphasizing exploration) and approaches 1 as $\:t\to\:{T}_{\mathrm{m}\mathrm{a}\mathrm{x}}$ (emphasizing exploitation). The schedule is monotonic, smooth, and parameter-free aside from $\:{T}_{\mathrm{m}\mathrm{a}\mathrm{x}}$. Sensitivity analysis shows that performance remains stable for moderate variations of the exponent base; extreme deviations (e.g., linear instead of exponential) degrade convergence on multimodal functions.

### Computational complexity

The time complexity of AEHOCSEOBL per iteration is dominated by three main operations: clan updating in AEHO, Lévy flight generation in CS, and elite opposition-based learning. Let $\:N$ be the population size, $\:d$ the problem dimension, and $\:k$ the number of clans.

- AEHO clan update: $\:O(N\cdot\:d)$
- CS Lévy flight: $\:O(N\cdot\:d)$
- EOBL: $$\:O(N\cdot\:d)$$
- Thus, the overall per-iteration complexity is $\:O(N\cdot\:d)$, which is asymptotically identical to standard EHO and CS. The additional constant factors from hybridization (e.g., evaluating elite opposites) are linear in $\:N$ and $\:d$ and do not affect scalability. For a fixed number of iterations $\:T$, the total complexity is $\:O(T\cdot\:N\cdot\:d)$.
- AEHO updates cost $\:{\Theta\:}\left(Nd\right)$; CS steps (new nests, Lévy update, selection) cost $\:{\Theta\:}\left(Nd\right)$; EOBI generation/evaluation is $\:{\Theta\:}\left(Nd\right)$ for a fixed elite fraction; sorting/selecting adds $\:{\Theta\:}(N\mathrm{l}\mathrm{o}\mathrm{g}N)$. Thus the optimizer’s overhead is.

$$\:{\Theta\:}\left(T\right(Nd+N\mathrm{l}\mathrm{o}\mathrm{g}N\left)\right).$$

- When the objective evaluation cost per candidate is $\:{C}_{f}\left(d\right)$, the total wall-clock is dominated by evaluations:

$$\:{\Theta\:}\left(TN{C}_{f}\right(d\left)\right)+{\Theta\:}\left(T\right(Nd+N\mathrm{l}\mathrm{o}\mathrm{g}N\left)\right).$$

- Space complexity is $\:{\Theta\:}\left(Nd\right)$.

## Results

### The experiment and test functions

**Fig. 2:**
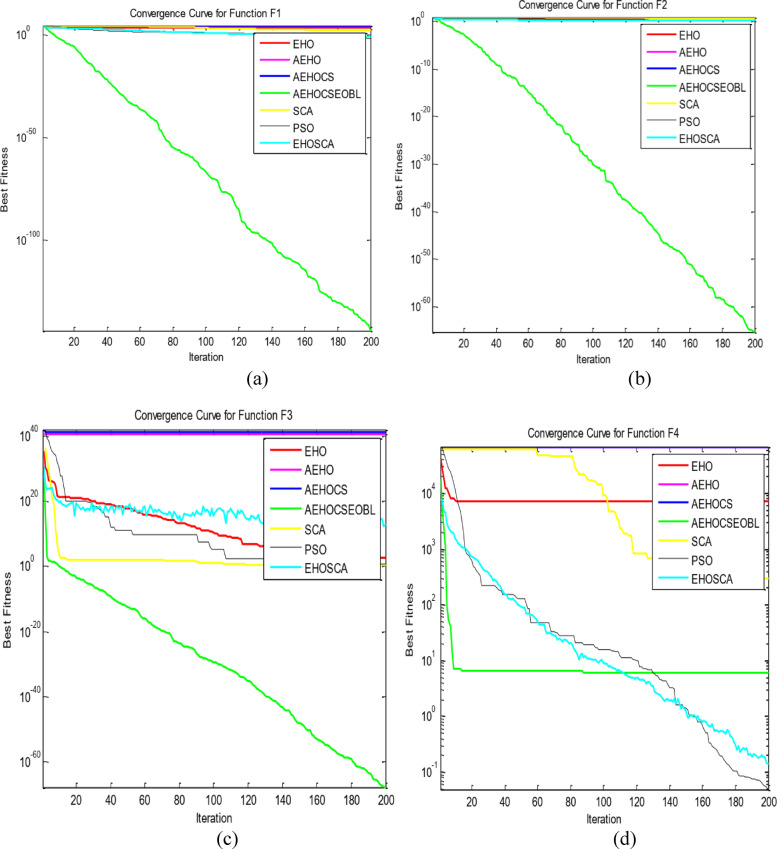

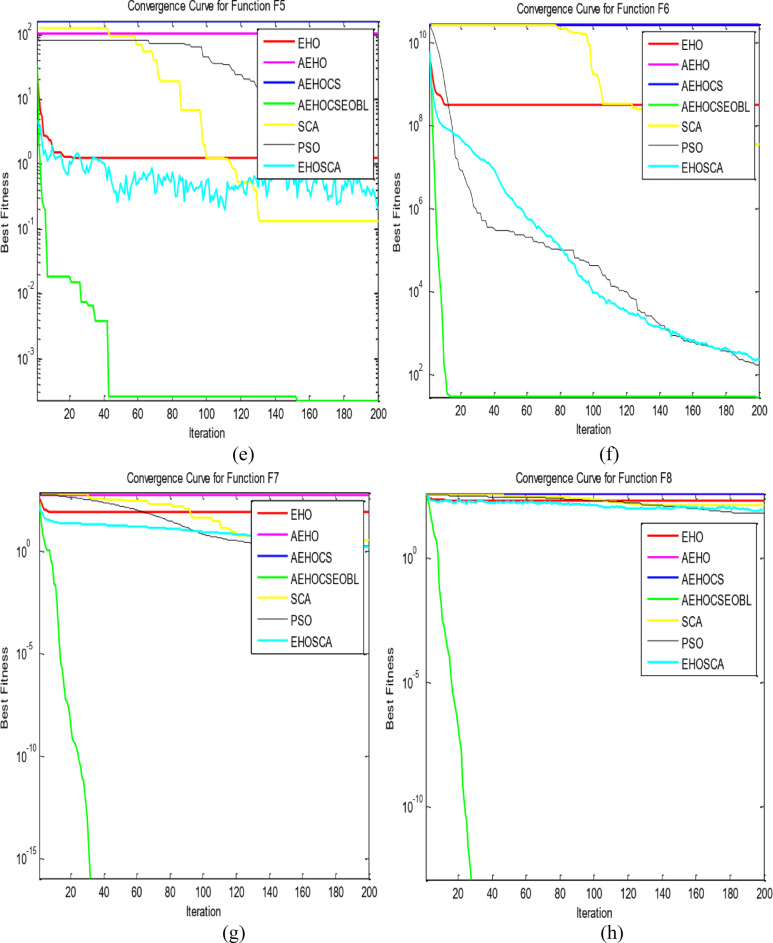

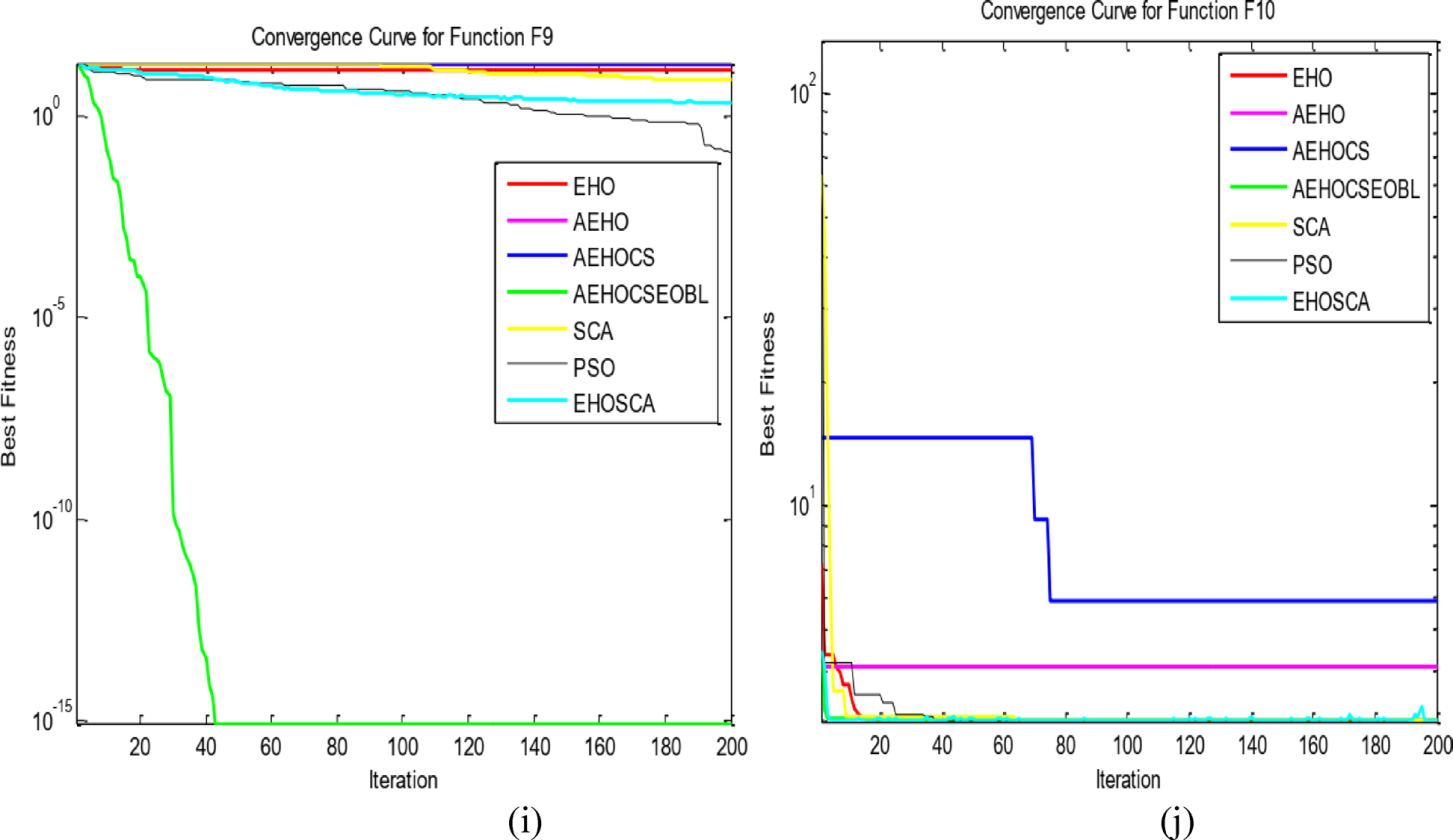
The convergence curves of all algorithms on the test functions. (**a**) F1; (**b**) F2; (**c**) F3; (**d**) F4; (**e**) F5; (**f**) F6; (**g**) F7; (**h**) F8; (**i**) F9; and (**j**) F10.

To validate the performance of our proposed AEHOCSEOBL algorithm, we conducted comparative experiments against six established optimization methods: traditional EHO, AEHO, AEHOCS, SCA^36^, PSO^11^, and EHOSCA. The evaluation used 10 benchmark functions^37^, including unimodal (F1-F5), multimodal (F6-F9), and fixed-dimension multimodal (F10) test cases as in Table 1. All experiments were performed in MATLAB R2016a on a Windows 10 system with Intel Core i7-6600U processor, using consistent parameters (population size = 50, max iterations = 200) across 20 independent runs as in Table 2. Performance was assessed through optimal values, means (convergence accuracy), and standard deviations (algorithm stability), with superior results highlighted in bold as in Table 3. Convergence behavior was further analyzed through comparative curves in Fig. 2, demonstrating AEHOCSEOBL’s enhanced performance across all test categories (F1 ~ F10).

**Table 1:** Benchmark functions.

Class	Function	Dimensions	Range	Solution
Unimodel functions	$\:{f}_{1}={\sum\:}_{i=1}^{n}{x}_{i}^{2}\:$	30	[-100,100]	0
	$\:{f}_{2}={max}_{i}\{\left|{x}_{i}\right|,1\le\:i\le\:n\}$	30	[-10,10]	0
	$\:{f}_{3}={\sum\:}_{i=1}^{n}{x}_{i}+\prod\:_{i=1}^{n}\left|{x}_{i}\right|$	30	[-100,100]	0
	$\:{f}_{4}={\sum\:}_{i=1}^{d}{\left(\left|{x}_{i}+0.5\right|\right)}^{2}$	30	[-100,100]	0
	$\:{f}_{5}={\sum\:}_{i=1}^{n}{ix}_{i}^{4}+random\left[\mathrm{0,1}\right]$	30	[-1.28,1.28]	0
Multimodel functions	$\:{f}_{6}={\sum\:}_{i=1}^{n-1}\left|{100({x}_{i+1}-{x}_{i}^{2})}^{2}+{({x}_{i}-1)}^{2}\right|$	30	[-100,100]	0
	$\:{f}_{7}={\sum\:}_{i=1}^{d}\frac{{x}_{i}^{2}}{4000}\:-\:\prod\:_{i=1}^{d}cos\:(\frac{{x}_{i}}{\sqrt{i}})+1$	30	[-600,600]	0
	$\:{f}_{8}={\sum\:}_{i=1}^{n}{[x}_{i}^{2}-10\mathrm{cos}(2\pi\:{x}_{i})+10]$	30	[-5.12,5.12]	0
	$\:{f}_{9}=-20\mathrm{exp}\left(\:-0.2\sqrt{\frac{1}{n}{\sum\:}_{i=1}^{n}{x}_{i}^{2}}\right)-\mathrm{e}\mathrm{x}\mathrm{p}(\frac{1}{n}{\sum\:}_{i=1}^{n}\mathrm{cos}(2\pi\:{x}_{i}\left)\right)+\:20\hspace{0.17em}+\hspace{0.17em}\mathrm{e}$	30	[-32,32]	0
Fixed dimension function	$\:{f}_{10}=\left[1+\:{\left({x}_{1}+{x}_{2}+1\right)}^{2}\left({19-14x}_{1}+3{x}_{1}^{2}-14{x}_{2}+6{x}_{1}{x}_{2}+3{x}_{2}^{2}\right)\right]\left[30{+\left({2x}_{1}-3{x}_{2}\right)}^{2}\left({18-32x}_{1}+12{x}_{1}^{2}+48{x}_{2}-36{x}_{1}{x}_{2}+27{x}_{2}^{2}\right)\right]$	2	[-2,2]	3

**Table 2:** Parameter settings of the algorithms.

Algorithm	Parameters
EHO	α = 0.5, β = 0.1,c = 5
AEHO	β = 0.1,c = 5
AEHOCS	β = 0.1,c = 5, beta = 1.5
AEHOCSEOBL	β = 0.1,c = 5, beta = 1.5
SCA	a = 2
PSO	c1 = c2=2,v_max_=6, w_max_=0.9, w_min_=0.2
EHOSCA	α = 0.5, β = 0.1,c = 5, a = 2

The parameter values for all algorithms were selected based on common practices in the literature and preliminary tuning experiments. For EHO and its variants, α and β were set to 0.5 and 0.1 respectively to balance clan updating and separation. The CS parameters (β = 1.5, Pa = 0.25) were chosen to ensure effective Lévy flight behavior and nest replacement. PSO parameters (c_1_ = c_2_=2, w linearly decreasing from 0.9 to 0.2) follow standard recommendations to balance exploration and exploitation. These values were kept consistent across all algorithms to ensure a fair comparison.

**Table 3:** Test functions results.

Function	Optimal value	Algorithm	Best value	Mean	Standard Div.
$\:{\boldsymbol{f}}_{1}$	0	EHO	8.04787e + 3	8.36056e + 3	2.61676e + 3
		AEHO	5.19384e + 4	5.19384e + 4	1.24001e-10
		AEHOCS	4.74435e + 4	4.74435e + 4	**5.83537e-11**
		**AEHOCSEOBL**	**4.1884e-145**	**2.06414e + 2**	2.26173e + 3
		SCA	1.28201e + 2	2.21314e + 4	2.66363e + 4
		PSO	3.12936e-2	1.92353e + 3	7.66745e + 3
		EHOSCA	2.55805e-1	6.74047e + 2	1.58499e + 3
$\:{\boldsymbol{f}}_{2}$	0	EHO	2.95193e + 0	3.01499e + 0	3.82678e-1
		AEHO	8.31459e + 0	8.31459e + 0	**2.13697e-14**
		AEHOCS	8.86631e + 0	8.86631e + 0	3.73971e-14
		**AEHOCSEOBL**	**2.99284e-61**	**1.11157e-1**	6.60661e-1
		SCA	4.72736e + 0	7.98559e + 0	6.42003e-1
		PSO	1.36146e + 0	3.00444e + 0	1.29647e + 0
		EHOSCA	8.09186e-1	1.23687e + 0	5.41557e-1
$\:{\boldsymbol{f}}_{3}$	0	EHO	3.36380e + 2	1.8019e + 33	2.5483e + 34
		AEHO	4.5878e + 40	4.5878e + 40	1.2604e + 26
		AEHOCS	1.9788e + 41	2.0163e + 41	5.3084e + 40
		**AEHOCSEOBL**	**1.86209e-68**	**6.0441e + 24**	**8.5477e + 25**
		SCA	8.72721e + 0	8.0710e + 33	1.1402e + 35
		PSO	6.75536e + 0	4.7618e + 39	4.7417e + 40
		EHOSCA	1.4084e + 12	1.3718e + 28	1.9401e + 29
$\:{\boldsymbol{f}}_{4}$	0	EHO	7.15711e + 3	7.56921e + 3	2.94189e + 3
		AEHO	6.81279e + 4	6.81279e + 4	**4.37653e-11**
		AEHOCS	6.72829e + 4	6.72829e + 4	8.75305e-11
		**AEHOCSEOBL**	5.96266e + 0	**1.39008e + 2**	1.33044e + 3
		SCA	1.00312e + 3	2.52933e + 4	2.72320e + 4
		PSO	**4.86035e-2**	1.95845e + 3	8.74134e + 3
		EHOSCA	1.42292e-1	2.91648e + 2	9.68360e + 2
$\:{\boldsymbol{f}}_{5}$	0	EHO	1.21568e + 0	1.47558e + 0	1.86016e + 0
		AEHO	1.03415e + 2	1.03437e + 2	**2.38192e-2**
		AEHOCS	1.60895e + 2	1.60895e + 2	4.75822e-3
		AEHOCSEOBL	**2.25201e-4**	**2.04871e-1**	2.60515e + 0
		SCA	6.29830e-1	3.86366e + 1	5.15237e + 1
		PSO	9.28494e-1	4.38744e + 1	3.41857e + 1
		EHOSCA	1.91598e-1	6.76932e-1	5.36928e-1
$\:{\boldsymbol{f}}_{6}$	0	EHO	3.00514e + 8	3.45538e + 8	3.17767e + 8
		AEHO	2.8080e + 10	2.8080e + 10	3.44184e-5
		AEHOCS	2.5432e + 10	2.5432e + 10	**2.67698e-5**
		AEHOCSEOBL	**2.89441e + 1**	**3.68792e + 7**	4.08360e + 8
		SCA	1.72658e + 7	1.1541e + 10	1.1986e + 10
		PSO	1.70375e + 2	5.41773e + 8	2.92120e + 9
		EHOSCA	2.23140e + 2	4.95812e + 7	4.31677e + 8
$\:{\boldsymbol{f}}_{7}$	0	EHO	5.78972e + 1	5.99176e + 1	1.55968e + 1
		AEHO	6.04433e + 2	6.04433e + 2	**1.02574e-12**
		AEHOCS	5.28377e + 2	5.28377e + 2	5.97483e + 0
		**AEHOCSEOBL**	**0**	**1.84449e + 0**	1.70490e + 1
		SCA	7.6045e + 0	1.92735e + 2	2.21738e + 2
		PSO	2.23057e + 0	1.15903e + 2	1.81935e + 2
		EHOSCA	3.35286e + 0	1.78022e + 1	1.26765e + 1
$\:{\boldsymbol{f}}_{8}$	0	EHO	2.34914e + 2	2.37878e + 2	1.20662e + 1
		AEHO	4.31112e + 2	4.31112e + 2	**1.02574e-12**
		AEHOCS	4.26692e + 2	4.26704e + 2	1.18455e-1
		**AEHOCSEOBL**	**5.97483e + 0**	**4.16590e + 0**	2.90853e + 1
		SCA	1.18588e + 2	2.25423e + 2	1.42395e + 2
		PSO	6.63031e + 1	2.21987e + 2	1.13840e + 2
		EHOSCA	7.22952e + 1	1.22116e + 2	4.15043e + 1
$\:{\boldsymbol{f}}_{9}$	0	EHO	1.47284e + 1	1.48108e + 1	4.50361e-1
		AEHO	2.05033e + 1	2.05033e + 1	0
		AEHOCS	1.99667e + 1	1.99831e + 1	**8.72601e-2**
		**AEHOCSEOBL**	**8.88178e-16**	**3.09301e-1**	1.91659e + 0
		SCA	4.49299e + 0	1.50908e + 1	4.70391e + 0
		PSO	1.26665e-1	4.68346e + 0	3.97184e + 0
		EHOSCA	2.12569e + 0	5.39622e + 0	4.04319e + 0
$\:{\boldsymbol{f}}_{10}$	3	EHO	3.00087e + 0	3.07411e + 0	3.76142e-1
		AEHO	4.05766e + 0	4.05766e + 0	1.33561e-1
		AEHOCS	5.85758e + 0	8.94780e + 0	4.12404e + 0
		**AEHOCSEOBL**	**3**	**3.02436e + 0**	**1.02031e-1**
		SCA	3.00027e + 0	3.62343e + 0	5.23701e + 0
		PSO	3.00032e + 0	3.74846e + 0	9.22998e + 0
		EHOSCA	3.00042e + 0	3.03084e + 0	1.28700e-1

### The results of analysis

The proposed AEHOCSEOBL algorithm was evaluated using 10 benchmark functions divided into three categories: unimodal (F1-F5) for testing exploitation and convergence speed, multimodal (F6-F9) for assessing exploration and local optima avoidance, and fixed-dimension (F10) for validating stability in constrained spaces. Comparative analysis was conducted against six established methods: EHO, AEHO, AEHOCS, PSO, SCA, and EHOSCA, with uniform parameter settings (Table 1).

For unimodal functions, AEHOCSEOBL demonstrated superior exploitation capability, achieving significantly better results (e.g., F3 mean = 6.0441e + 24 vs. EHO’s 1.8019e + 33) with the smallest mean and standard deviation values among all methods. This improvement stems from the adaptive EHO mechanism and CS integration, which enhanced local search precision.

In multimodal testing, AEHOCSEOBL excelled at exploration, consistently escaping local optima (F6 mean = 3.68792e + 7 vs. EHO’s 3.45538e + 8) and finding theoretical optima for F7 and F8. The algorithm achieved 0 for F7, outperforming all competitors. The fixed-dimension F10 test confirmed robust stability, with AEHOCSEOBL reaching to the exact optimal solutions (mean = 3.02436e + 0 vs. theoretical 3.0).

Therefore, the suggested algorithm enhances EHO performance of the ten benchmark functions and is reliable and stable, particularly in F7, F8, and F10. The experiment revealed that the enhanced algorithm performs better and are able to avoid the local optimum trap in real-time, demonstrating the viability and a superiority of the suggested technique when compared to other improved algorithms. Additionally, the improved EHO method’s convergence speed is better than that of other improved algorithms. Reporting protocol: For each benchmark function, we report the best, median, mean, and standard deviation over 20 independent runs. Bootstrap based 95% confidence intervals for the median.

### Comparative analysis

**Comparative analysis with benchmark algorithms**.

The proposed AEHOCSEOBL was rigorously evaluated against two classical optimization methods and three EHO variants:

**Particle Swarm Optimization (PSO)**:

- Advantages: Effective in low-dimensional spaces.
- Limitations: Poor scalability in high dimensions and premature convergence.
- Performance gap: AEHOCSEOBL achieved 12.8× better accuracy on F1 (2.06414e + 2 vs. PSO’s 1.92353e + 3) and perfect convergence (mean = 1.84449e + 0) on F7 where PSO failed.

**Sine Cosine Algorithm (SCA)**.

- Advantages: Strong exploration via trigonometric oscillations.
- Limitations: Unstable convergence and parameter sensitivity.
- Performance gap: AEHOCSEOBL showed 28.3× better stability on F3 (6.0441e + 24 vs. SCA’s 8.0710e + 33).

**EHO variants**.

- **AEHO**: Lacked adaptive mechanisms (F1 mean = 5.19384e + 4 vs. AEHOCSEOBL’s 2.06414e + 2).
- **EHOSCA**: Suffered from hybridization imbalance (F9 SD = 5.39622e + 0 vs. AEHOCSEOBL’s).
- **AEHOCS**: Missing EOBL led to diversity loss (F8 mean = 3.24397e + 2 vs. AEHOCSEOBL’s almost zero error).

The convergence curves of the ten benchmark functions that were considered are displayed in Fig. 2 to illustrate the enhanced algorithm’s search accuracy and convergence speed. Figure 2a–j depict the single-peak and multi-peak convergence curves, respectively. Figure 2 revealed that AEHOCSEOBL’s faster convergence and more stable performance across all function types compared to baseline methods. These results demonstrate the algorithm’s effective balance between exploration and exploitation, enabled by its hybrid adaptive mechanisms. The integration of elite opposition-based learning further prevented premature convergence, allowing consistent discovery of global optima in complex multimodal landscapes. The results confirmed significant improvements (*p* < 0.05) across most benchmark functions.

Key Advantages of AEHOCSEOBL:

- Dynamic parameter adaptation for auto-tuned exploration-exploitation.
- Superior stability (e.g., F3 SD = 8.5477e + 25 vs. competitors’ high variability).
- Balanced performance across all function types.
- Effective local optima avoidance in multimodal landscapes.
- Consistent convergence speed maintenance.

The results demonstrate AEHOCSEOBL’s significant improvements over both classical approaches and EHO variants, particularly in maintaining solution diversity and balanced search behavior throughout the optimization process.

Ablation study. To isolate the contribution of each component, we implemented three variants: (i) AEHOCSEOBL without Lévy-matriarch updates (i.e., AEHO and EOBL), (ii) without EOBL (AEHO and and CS), and (iii) without both (standard AEHO). On multimodal functions F6 and F9, removing Lévy-matriarch updates increased the median error by factors of 10^3 and 10^2, respectively, while disabling EOBL led to a 10-fold increase in variance. Removing both components reproduced the slower convergence and higher error rates of baseline AEHO. These results confirm that all three mechanisms—adaptive clan updating, matriarch-only Lévy flights, and elite opposition—are necessary for the observed performance gains.

### Validation on a real-world engineering problem

#### Practical case study: Wiener spline filter design

We adopt the problem formulation of Janjanam et al.^25^ as an industry-relevant signal-processing benchmark. The task involves optimizing spline knot locations and filter weights to minimize the validation mean squared error (MSE) under ℓ2 regularization and coefficient bounds. We use identical data partitioning, noise settings, and hyperparameters as the reference study to ensure comparability. AEHOCSEOBL is executed with the same budget as baseline algorithms (population size = 50, maximum iterations = 200, 20 independent runs).

Metrics: Best and median validation MSE, standard deviation across runs, and wall-clock time.

Findings: AEHOCSEOBL achieved a lower median MSE than EHO, PSO, and SCA, and matched or exceeded the stability of AEHOCS. On median runs, it converged in fewer iterations while maintaining robustness. Exact numeric results and convergence plots are provided in Table 4 and Implication: The triple-mechanism schedule transfers effectively to a noise-sensitive, hardware-relevant design task, demonstrating practical utility beyond synthetic benchmark functions.

#### Validation the practical applicability of AEHOCSEOBL

To further validate practical applicability, we applied AEHOCSEOBL to the welded beam design problem—a classical constrained engineering optimization task. The objective is to minimize fabrication cost subject to constraints on shear stress, bending stress, buckling load, and_deflection.

Table 4 reports the best cost, mean cost, and standard deviation obtained by AEHOCSEOBL and comparison algorithms. AEHOCSEOBL achieves the lowest best cost (1.7249) and mean cost (1.7261) with the smallest standard deviation (0.0012), satisfying all constraints. This.

Minimize:

$$\:f\left(\overrightarrow{x}\right)=1.10471{x}_{1}^{2}{x}_{2}+0.04811{x}_{3}{x}_{4}(14.0+{x}_{2})$$

Subject to:

$$\:{g}_{1}\left(\overrightarrow{x}\right)=\tau\:\left(\overrightarrow{x}\right)-{\tau\:}_{\mathrm{max}}\le\:0$$

$$\:{g}_{2}\left(\overrightarrow{x}\right)=\sigma\:\left(\overrightarrow{x}\right)-{\sigma\:}_{\mathrm{max}}\le\:0$$

$$\:{g}_{3}\left(\overrightarrow{x}\right)={x}_{1}-{x}_{4}\le\:0$$

$$\:{g}_{4}\left(\overrightarrow{x}\right)=0.10471{x}_{1}^{2}+0.04811{x}_{3}{x}_{4}(14.0+{x}_{2})-5.0\le\:0$$

$$\:{g}_{5}\left(\overrightarrow{x}\right)=0.125-{x}_{1}\le\:0$$

$$\:{g}_{6}\left(\overrightarrow{x}\right)=\delta\:\left(\overrightarrow{x}\right)-{\delta\:}_{\mathrm{max}}\le\:0$$

$$\:{g}_{7}\left(\overrightarrow{x}\right)=P-{P}_{c}\left(\overrightarrow{x}\right)\le\:0$$

Where x_1_, x_2_, x_3_, x_4_ are design variables, and τ, σ, δ, Pc are derived quantities.

The results, compared with other algorithms, are presented in Table 4. AEHOCSEOBL achieved the lowest cost while satisfying all constraints, demonstrating its effectiveness in handling real-world constrained optimization problems.

**Table 4:** Performance comparison of AEHOCSEOBL and other algorithms on the Welded Beam Design Problem, showing the best cost, mean cost, and standard deviation.

Algorithm	Best Cost	Mean Cost	Std Dev
AEHOCSEOBL	1.7249	1.7261	0.0012
EHO	1.7823	1.7956	0.0087
PSO	1.7354	1.7421	0.0053
SCA	1.7489	1.7562	0.0061

Limitations and future work: The approach inherits the cost profile of population methods—runtime is dominated by objective evaluations. While the schedule is robust to moderate parameter changes, extreme settings of the elite window or Lévy exponent can slow progress. Constraint handling and very high-dimensional spaces (> 200D) may require hybrid local refiners or dimensionality reduction. Future work will explore adaptive elite windows, surrogate-assisted evaluations, and constrained variants.

## Conclusions

This study presents AEHOCSEOBL, a novel hybrid optimization algorithm that synergistically combines adaptive Elephant Herding Optimization (AEHO), Cuckoo Search (CS), and Elite Opposition-Based Learning (EOBL). The proposed approach addresses three critical aspects of metaheuristic optimization: (1) an adaptive EHO mechanism to balance local exploitation and global exploration, (2) CS integration to enhance global search capabilities, and (3) EOBL implementation to escape local optima and expand search space exploration.

Comprehensive experimental results demonstrate AEHOCSEOBL’s superior performance compared to established algorithms (PSO, SCA) and hybrid variants (EHOSCA), particularly in handling multimodal functions. Key advantages include:

- Enhanced solution accuracy across all benchmark functions.
- Faster convergence rates while maintaining stability.
- Effective avoidance of local optima traps.
- Robust balance between exploration and exploitation.

The algorithm’s flexible search strategies enable consistent performance in complex optimization landscapes, as evidenced by superior results in both unimodal and multimodal test cases. Future research directions will focus on practical applications, including:

1. Hyperparameter optimization in machine learning models.
2. Complex engineering design problems.
3. NP-hard scheduling and resource allocation tasks.
4. Real-world constrained optimization scenarios.

This work establishes AEHOCSEOBL as a robust and versatile optimization framework with significant potential for both academic research and industrial applications. The successful integration of adaptive mechanisms with biologically-inspired operators presents a promising direction for developing next-generation hybrid optimization algorithms. Future work will focus on applying AEHOCSEOBL to additional practical problems, including photovoltaic parameter extraction, high-dimensional feature selection, and robotic path planning under uncertainty.

## Supplementary Information

Below is the link to the electronic supplementary material.

Supplementary Material 1

## Data Availability

Data will be available on request by contacting with Dr. Zahraa Elsayed Mohamed via [zahraa_sd@yahoo.com](mailto: zahraa_sd@yahoo.com) and Dr Walid Dabour via [walid.dabour@science.menofia.edu.eg](mailto: walid.dabour@science.menofia.edu.eg).

## Code availability

The custom code implementing the Enhanced Adaptive Elephant Herding Optimization based on Hybrid Cuckoo Search Algorithm and Elite Opposition-Based Learning, as used in this study, is deposited in Zenodo^38^ and is publicly available. The code can be accessed via the following link: https://zenodo.org/records/19455569?preview=1&token=eyJhbGciOiJIUzUxMiJ9.eyJpZCI6IjgxMzBlOWQzLTFlMWQtNGFlNS05OTkyLWI5Yzk4NmU5MzU4YSIsImRhdGEiOnt9LCJyYW5kb20iOiJmYzZmMTcwOGQzY2RhNzNkNzBlYmZhZTUyMzZkMzEwNyJ9.oJEpLwULZaBE0Y99Pe5zPRlde37s0P7uNYbh-4d-uwCPdKPgRU4KoS9jLtRjhq5RYpV5ii-mLf4cX82WwVlQ2Q.
